# Potential role of the lectin pathway of complement in the pathogenesis and disease manifestations of systemic sclerosis: a case-control and cohort study

**DOI:** 10.1186/s13075-014-0480-6

**Published:** 2014-11-18

**Authors:** Michael Osthoff, Gene-Siew Ngian, Melinda M Dean, Mandana Nikpour, Wendy Stevens, Susanna Proudman, Damon P Eisen, Joanne Sahhar

**Affiliations:** Victorian Infectious Diseases Service, Royal Melbourne Hospital, Grattan Street, Parkville, VIC 3050 Australia; Department of Medicine, The University of Melbourne, Grattan Street, Melbourne, VIC 3010 Australia; Department of Rheumatology, Monash Medical Centre, 246 Clayton Road, Clayton, VIC 3168 Australia; Department of Rheumatology, Royal Melbourne Hospital, Grattan Street, Parkville, VIC 3050 Australia; Research and Development, Australian Red Cross Blood Service, 44 Musk Avenue, Brisbane, QLD 4059 Australia; Department of Rheumatology, St. Vincent’s Hospital, 41 Victoria Parade, Melbourne, VIC 3065 Australia; Department of Medicine, The University of Melbourne at St. Vincent’s Hospital, 41 Victoria Parade, Melbourne, VIC 3065 Australia; Rheumatology Unit, Royal Adelaide Hospital, North Terrace, Adelaide, SA 5000 Australia; Discipline of Medicine, University of Adelaide, North Terrace, Adelaide, SA 5000 Australia; Monash University, Wellington Road, Clayton, VIC 3168 Australia

## Abstract

**Introduction:**

Repetitive episodes of ischemia and reperfusion (I/R) are a cardinal feature of the pathogenesis of systemic sclerosis (SSc), which precedes tissue fibrosis. The complement system is a key mediator of tissue damage after I/R, primarily by activation of the lectin pathway. This study investigated whether serum levels and polymorphisms of mannose-binding lectin (MBL) and ficolin-2 (FCN2), two pattern recognition receptors of the lectin pathway, are associated with the predisposition to and clinical features of SSc.

**Methods:**

A case-control study was undertaken involving 90 patients with SSc from a single SSc outpatient clinic and 90 age- and sex-matched blood donors. MBL and FCN2 levels and polymorphisms were measured in both groups, and in cases correlated with clinical data.

**Results:**

MBL levels and genotypes were equally distributed in cases and controls while there were some significant differences in *FCN2* polymorphisms. Median MBL levels were higher in SSc cases with diffuse disease compared with controls (2.6 versus 1.0 μg/ml, *P* <0.001).

In cases, higher MBL levels were associated with the presence of clinical findings associated with vascular dysfunction and local tissue damage (digital ulcers, calcinosis and pitting). Moreover, MBL levels were associated with fibrotic disease manifestations as evidenced by the presence of diffuse disease (median 2.6 versus 0.8 μg/ml, *P* = 0.002), the modified Rodnan skin score (r = 0.39, *P* <0.001), and interstitial lung disease as measured by forced vital capacity (r = −0.33, *P* = 0.001). Importantly, MBL levels also correlated with the Scleroderma Health Assessment Questionnaire scores (r = 0.33, *P* = 0.002). The results for FCN2 levels were less striking. Phenotypic MBL results were largely confirmed by analysis of MBL polymorphisms. MBL levels were not associated with the presence of autoantibodies or hypocomplementaemia.

**Conclusions:**

Overall, predisposition to SSc was not influenced by the lectin pathway of complement in our matched case-control study. However, our preliminary data suggest that MBL, and to a lesser extent FCN2, may modulate disease manifestations of SSc, particularly in diffuse cutaneous disease.

**Electronic supplementary material:**

The online version of this article (doi:10.1186/s13075-014-0480-6) contains supplementary material, which is available to authorized users.

## Introduction

Systemic sclerosis (SSc) is a complex autoimmune disease associated with a high morbidity and mortality [1], in which vascular injury, extensive fibrosis, and autoantibodies are the cardinal pathologic features. The extent of cutaneous fibrosis is used to classify SSc patients into two major subgroups: limited cutaneous and diffuse cutaneous SSc [2]. Genetic factors have been demonstrated to influence disease susceptibility as well as patterns of disease expression [3]. However, the pathogenesis of SSc is complex and remains incompletely understood with much of the research having been focused on fibroblast activation and fibrosis rather than vascular dysfunction. Intriguingly, the concept of vascular injury as a fundamental element in the pathogenesis of SSc was suggested more than 120 years ago [4]. Recent evidence supports the hypothesis that microvascular injury is an early event in the pathogenesis of SSc which precedes tissue fibrosis [5]. Initially, an undetermined triggering event causes mild oxidative stress, coinciding with perivascular inflammation and endothelial dysfunction. Repetitive episodes of ischemia and reperfusion (I/R) and persistent oxidative stress are responsible for progressive endothelial dysfunction, activation of vascular mesenchymal cells and leakage of growth factors leading to progressive tissue fibrosis and finally late organ dysfunction [6]. Importantly, vascular damage occurs in virtually all organs with cardiopulmonary involvement being responsible for the majority of disease-related mortality [7]. Later in the disease, inflammation subsides with atrophy and organ derangement prevailing. Apart from the adaptive immune system, components of innate immunity have been implicated in the pathogenesis of SSc including the complement system, an ancient cascade of circulating serum proteins and proteases [8,9].

The lectin pathway of complement is activated after binding of mannose-binding lectin (MBL) or ficolins (ficolin-1, -2, and -3) to carbohydrate patterns, acetyl groups or immunoglobulin M (IgM) bound to antigens on pathogens and dying cells, with subsequent activation of MBL-associated serine protease (MASP)-1 and -2 and assembly of the C3 convertase [10]. Interestingly, a direct link between the MBL-MASP complex and the coagulation cascade has been recently shown without the involvement of downstream complement components [11,12]. The serum concentration of these pattern recognition receptors (PRR) vary between individuals, with MBL showing the greatest difference (from undetectable to about 10 μg/mL), as a result of polymorphisms in the exon and promoter region of the *MBL2* gene on chromosome 10 [13]. Notably, the prevalence of moderate to severe MBL deficiency is as high as 30% in most populations investigated to date [13]. Similarly, a number of major polymorphisms have been described for the ficolin-2 gene (*FCN2*) with a much weaker genotypic/phenotypic relationship than is the case for MBL [14].

MBL deficiency has been implicated as a risk factor in other autoimmune diseases such as systemic lupus erythematosus [15]. In addition, several recent studies have acknowledged the important role of MBL and ficolin-2 in I/R injury [16,17]. MBL and the lectin complement pathway seem to be an essential component of the inflammatory process augmenting I/R injury in various organs, such as the heart and the brain [18,19]. Conversely, blockade of MBL and lectin pathway activation results in diminished tissue injury [18,20,21]. In line, human MBL deficiency was found to be associated with a significant reduction in morbidity and mortality after acute myocardial infarction or acute ischemic stroke, respectively [22,23].

Evidence for an involvement of MBL or the lectin pathway in the pathogenesis of SSc is lacking. To address this question, we investigated the association of serum levels and polymorphisms of two PRRs of the lectin pathway, MBL and ficolin-2, with predisposition to and clinical features of SSc. Given the importance of vascular I/R injury in SSc on the one hand, and the central involvement of the lectin pathway in I/R injury on the other hand, we hypothesized that the presence of MBL deficiency and polymorphisms in the *MBL2* and *FCN2* genes are associated with a reduced risk of SSc and less severe disease manifestations in SSc patients.

## Methods

### Participants

We conducted a matched case-control and cross-sectional study involving 90 patients with SSc or mixed connective tissue disease (MCTD) from a single SSc outpatient clinic (Monash Health) and 90 age- and sex-matched blood donors. This study has been approved by the Human Research and Ethics Committee of Monash Health, Melbourne Health and the Australian Red Cross Blood Service.

Clinical and corresponding laboratory data and stored blood samples were available from SSc patients who had been previously recruited at a single SSc outpatient clinic as part of the Australian Scleroderma Cohort study (ASCS), an ongoing longitudinal cohort study of predictive factors for the development of cardiopulmonary complications and outcomes of therapy in SSc. Participants had consented to collection, storage and use of blood samples and data for future SSc-related research projects at inclusion into the ASCS. Age- (within five years) and sex-matched controls were selected from a previous cohort of 426 healthy blood donors (Australian Red Cross Blood Service (ARCBS), Brisbane, Australia) for whom informed consent was waived by the Human Research and Ethics Committee of the ARCBS (ethics approval 2011#08).

SSc patients were over 18 years of age with a clinical diagnosis of SSc according to the American College of Rheumatology criteria [24] or the LeRoy and Medsger criteria [25] and were classified using the LeRoy criteria for limited or diffuse SSc [2]. Patients were diagnosed with MCTD according to the Alarcon-Segovia criteria [26]. Clinical and laboratory data (that is C-reactive protein (CRP), creatinine, erythrocyte sedimentation rate (ESR), complement C3 and C4) and serum samples collected at the most recent annual visit (April 2012 till April 2013) were used for the purpose of this study. Clinical data included past medical history, past and current medication, current skin findings, organ involvement, results from pulmonary function testing, transthoracic echocardiogram and the six-minute walk test, modified Rodnan skin score (mRSS), EULAR Scleroderma Trial and Research Group (EUSTAR) Systemic Sclerosis Activity Score, and Scleroderma Health Assessment Questionnaire (SSc HAQ) score. Results from autoantibody screening were available from the first study visit.

Pulmonary arterial hypertension (PAH) was diagnosed based on right heart catheterisation findings of a mean pulmonary artery pressure of greater than 25 mm Hg at rest or greater than 30 mm Hg on exercise, in the presence of a pulmonary capillary wedge pressure of less than 18 mm Hg. Interstitial lung disease (ILD) was defined as the presence of characteristic abnormalities on high-resolution computed tomography (CT) chest scan, and scleroderma renal crisis was defined as the presence of at least two of: new-onset hypertension, microangiopathic anemia or rising creatinine. Immunosuppressive medication was defined as current use of prednisolone (>5 mg daily), methotrexate, azathioprine, mycophenolate mofetil, cyclosporine A, or cyclophosphamide. Disease duration was calculated from the onset of the first non-Raynaud’s phenomenon symptom.

### Definition of endpoints

The main aim of this study was to compare serum MBL levels and the frequency of MBL deficiency in SSc patients with age- and sex-matched controls. Additional aims included investigating the association of MBL and ficolin-2 levels and polymorphism with disease manifestations and autoantibodies in SSc cases only.

### Determination of MBL and ficolin-2 plasma levels

Quantification of MBL serum levels was performed by an investigator blinded to clinical data using a mannan-binding enzyme-linked immunosorbent assay (ELISA) as previously described [27]. Briefly, mannan-coated microtitre plates were incubated with samples at 1:25 and 1:100 dilutions for 90 minutes at room temperature followed by detection of bound MBL with a biotinylated monoclonal anti-MBL antibody (HYB 131-01, BioPorto Diagnostics, Hellerup, Denmark). MBL deficiency was defined as serum level <0.5 μg/mL. Ficolin-2 serum levels were quantified using a commercially available ELISA kit (Hycult, Udin, The Netherlands). As different kinds of analytic tubes were used in cases and controls, ficolin-2 levels were only determined in cases (with the same analytic tube used for all cases) in agreement with recent studies showing significant differences in ficolin-2 levels depending on the blood collection system [28,29].

### MBL2 and ficolin-2 genotyping

DNA lysates were prepared according to the manufacturer’s instructions (TaqMan Sample-to-SNP, Life Technologies, Mulgrave, VIC, Australia). Subsequently, *MBL2* and *FCN2* promoter and exon polymorphisms were determined by allele-specific polymerase chain reaction (PCR) using TaqMan fluorescent probes (TaqMan genotyping assays, Life Technologies) as described elsewhere [30]. For assay details, see Table S1 in Additional file 1.

*MBL2* genotypes were classified as low (XA/YO, YO/YO), intermediate (XA/XA, YA/YO) or high (YA/YA, XA/YA) producing genotypes according to published literature [31], with exon variant alleles collectively designated as O and the wild-type gene as A, and the promoter variant allele and the wild-type gene designated as X and Y, respectively. *FCN2* promoter and exon polymorphisms were analyzed separately and combined as haplotypes [32].

### Statistical analysis

We used the chi-square test for comparisons of categorical variables and allele and genotype frequencies and to check for Hardy-Weinberg equilibrium. To investigate MBL and ficolin-2 as potential risk factors for SSc, matched univariate analysis was performed by running conditional logistic regression on one variable at a time with SSc as the dependent variable. In addition, Wilcoxon signed-rank test was applied to compare MBL levels in cases and matched controls. Haplotype frequencies were analyzed by expectation-maximum algorithm.

Differences in disease severity and organ involvement according to MBL and ficolin-2 levels were analyzed using the Mann-Whitney *U* test (with reporting of median and interquartile range (IQR) due to the non-Gaussian distribution of MBL levels) and the Student’s *t* test (with reporting of mean and standard deviation (SD)), respectively. Continuous variables were correlated with MBL and ficolin-2 levels using the Spearman rank and Pearson correlation tests, respectively. Linear regression analysis was used to estimate the association of MBL levels with mRSS and SSc HAQ score after adjustment for age, sex, presence of ILD and PAH.

All testing was two-tailed. Haplotype and linkage disequilibrium analysis was carried out with the Haploview program (version 4.2, The Broad Institute, Cambridge, MA, USA). All other analyses were performed with SPSS statistical software, version 17.0 (SPSS Inc., Chicago, IL, USA).

## Results

### Demographic, clinical and lectin pathway characteristics of cases and controls

The study population consisted of 90 SSc cases and 90 age- and sex-matched blood donors, with population demographics and clinical characteristics outlined in Table 1. Mean age (SD) was 60 (15) and 59 (16) years in cases and controls, respectively, and 78 (87%) were female in both groups. Cases were classified as limited and diffuse SSc in 69% and 26%, respectively, with the remaining patients having been diagnosed with MCTD (4/90) or sine scleroderma (1/90). Anti-centromere antibodies were present in 38/90 cases and anti-Scl-70 antibodies in 17/90 cases. Apart from gastroesophageal reflux, ILD represented the most frequent organ involvement (41/90), with PAH, myocardial involvement and scleroderma renal crisis encountered less frequently.

**Table 1:** **Characteristics of cases and controls**

**Characteristics**	**Cases**	**Controls**
	**(n = 90)**	**(n = 90)**
Female, n (%)	78 (87)	78 (87)
Age (years), mean (SD)	60 (15)	59 (16)
*Race, n (%)*		
Caucasian	76 (84)	
Asian	9 (10)	
Other	5 (6)	
Duration of disease (years), mean (SD)	14.3 (10.3)	
*SSc subtype, n (%)*		
Diffuse disease	23 (26)	
Limited disease	62 (69)	
Sine	1 (1)	
Mixed connective tissue disease	4 (4)	
*Autoantibodies, n (%)*		
Anti-topoisomerase I (Scl-70)	*17 (19)*	
Anti-centromere	*38 (42)*	
Anti-RNA polymerase III	*8 (9)*	
*Organ involvement, n (%)*		
Bowel dysmotility	9 (10)	
Pulmonary arterial hypertension	8 (9)	
Interstitial lung disease	41 (46)	
Renal crisis	4 (4)	
Gastroesophageal reflux disease	83 (92)	
*Disease activity/severity*		
Active digital ulcers, n (%)	13 (14)	
mRSS, mean (SD)	8.9 (7.3)	
SSc HAQ, mean (SD)	20.5 (13.4)	
EUSTAR SSc activity score, mean (SD)	2.2 (1.7)	
Immunosuppressive agents, n (%)	28 (31)	
Past treatment with iloprost, n (%)	17 (19)	
FVC (% predicted), mean (SD)	91.5 (22.5)	
DLCO (% predicted), mean (SD)	58.5 (18.8)	
6MWD (meter), mean (SD) meters	471.7 (126.1)	

6MWD, six-minute walk distance; DLCO, diffusing capacity of the lung for carbon monoxide; EUSTAR, EULAR Scleroderma Trials and Research Group; FVC, forced vital capacity; mRSS, modified Rodnan skin score; SD, standard deviation; SSc, systemic sclerosis; SSc HAQ, Scleroderma Health Assessment Questionnaire.

**Figure 1:**
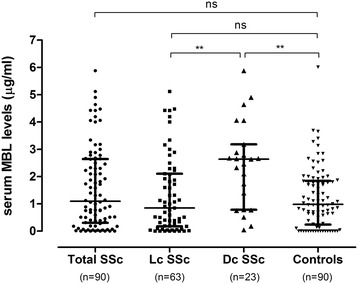
**Serum mannose-binding lectin levels in SSc cases and healthy controls.** MBL levels were analyzed in SSc cases overall and stratified according to skin involvement (limited cutaneous vs. diffuse cutaneous. Horizontal bars indicate median and 25 to 75 percentiles. Dc SSc, diffuse cutaneous systemic sclerosis; Lc SSc, limited cutaneous systemic sclerosis; MBL, mannose-binding lectin.

*MBL2* and *FCN2* allele frequencies at all 10 positions were in agreement with the predicted Hardy-Weinberg equilibrium (data not shown). The frequency of *MBL2* mutations at four positions, *MBL2* genotypes and MBL deficiency (defined as serum levels <0.5 μg/mL) was similar in SSc cases and controls, as were overall MBL serum levels (Table 2). However, MBL serum levels were significantly higher in diffuse SSc patients compared to limited SSc cases and controls (Figure 1). In line with this, MBL deficiency was rare in diffuse compared to limited SSc patients or controls (2 (9%) vs. 36 (39%) vs. 25 (28%), *P* = 0.02), as were low-producing *MBL2* genotypes (1(4%) vs. 17 (25%) vs. 18 (20%), *P* = 0.1).

**Table 2:** **Analysis of** ***MBL2*** **geno- and phenotypes in SSc cases and controls**

**Variables**	**Cases**	**Controls**	**Univariate matched analysis**	
	**(n = 90)**	**(n = 90)**	**OR (95% CI)**	***P*** **value**
*MBL2 exon variants, n (%)*				
A/A	56 (62)	59 (66)	Reference	
A/O	30 (33)	25 (28)	1.2 (0.7-2.3)	0.5
O/O	4 (4)	6 (7)	0.6 (0.1-3.1)	0.5
*MBL2 promoter variant, n (%)*				
Y/Y	46 (51)	47 (52)	Reference	
Y/X	36 (40)	38 (42)	1.0 (0.6-1.7)	1
X/X	8 (9)	5 (6)	1.7 (0.5-6.2	0.4
*MBL2 genotypes, n (%)*				
High producing	48 (53)	54 (60)	Reference	
Intermediate producing	24 (27)	18 (20)	1.5 (0.7-3.0)	0.3
Low producing	18 (20)	18 (20)	1.1 (0.5-2.5)	0.8
MBL levels (μg/ml), median (IQR)	1.1	1.0	1.3 (1.0-1.7)^a^	0.06
MBL <0.5 μg/ml, n (%)	62 (69)	65 (72)	1.2 (0.6-2.5)	0.6

*MBL2* genotypes were classified as low- (XA/YO, YO/YO), intermediate- (XA/XA, YA/YO) or high- (YA/YA, XA/YA) producing genotypes with exon variant alleles collectively designated as O and the wild-type gene as A, and the promoter variant allele and the wild-type gene designated as X and Y, respectively. ^a^Per 1 μg/ml increase in MBL serum levels. CI, confidence interval; IQR, interquartile range; MBL, mannose-binding lectin; OR, odds ratio; SSc, systemic sclerosis.

Regarding *FCN2* polymorphisms, there were only minor differences at two positions (*FCN2* -4 and +6359) in cases and controls (Table 3).

**Table 3:** **Analysis of** ***FCN2*** **polymorphisms in SSc cases and controls**

**Variables**	**Cases**	**Controls**	**Univariate matched analysis**	
	**(n = 90)**	**(n = 90)**	**OR (95% CI)**	***P*** **value**
*FCN2* promoter variants, n (%)				
*FCN2 -986, n (%)*				
G/G	27 (30)	22 (24)	Reference	
G/A	39 (43)	44 (49)	0.7 (0.3-1.5)	0.4
A/A	24 (27)	24 (27)	0.8 (0.4-1.7)	0.6
*FCN2 - 602, n (%)*				
G/G	57 (63)	63 (70)	Reference	
G/A	25 (28)	23 (26)	1.1 (0.6-2.3)	0.7
A/A	8 (9)	4 (4)	2.0 (0.6-6.7)	0.25
*FCN2 - 557, n (%)*				
A/A	65 (72)	76 (84)	Reference	
A/G	23 (26)	13 (14)	2.1 (1.0-4.7)	0.07
G/G	2 (2)	1 (1)	2 (0.2-22.0)	0.6
*FCN2 - 4, n (%)*				
A/A	53 (59)	41 (46)	Reference	
A/G	35 (39)	41 (46)	0.7 (0.4-1.2)	0.18
G/G	2 (2)	8 (9)	0.2 (0.04-0.99)	**0.048**
*FCN2* exon variants, n (%)				
*FCN2 + 6359, n (%)*				
C/C	49 (54)	40 (44)	Reference	
C/T	39 (44)	40 (44)	0.8 (0.4-1.4)	0.4
T/T	2 (2)	10 (11)	0.2 (0.04-0.8)	**0.03**
*FCN2 + 6424, n (%)*				
G/G	65 (72)	74 (82)	Reference	
G/T	22 (24)	15 (17)	1.6 (0.8-3.2)	0.2
T/T	3 (3)	1 (1)	3.4 (0.4-33.0)	0.3
*FCN2* haplotypes, n (%)				
GGAACG	31 (35)	37 (41)	Reference	
AGAGTG	18 (20)	26 (30)	0.6 (0.4-1.1)	0.1
AAAACG	19 (22)	14 (15)	1.4 (0.8-2.4)	0.3
GGGACT	11 (13)	7 (7)	1.7 (0.7-3.8)	0.2
AGAACG	4 (4)	2 (2)	1.7 (0.5-5.0)	0.4

CI, confidence interval; FCN2, ficolin-2; OR, odds ratio; SD, standard deviation; SSc, systemic sclerosis.

### Association of MBL and ficolin-2 with SSc disease activity, inflammatory markers and autoantibodies

Apart from MBL, mean ficolin-2 levels were also significantly higher in diffuse SSc patients compared to limited and sine scleroderma SSc patients (0.71 (0.38) vs. 0.51 (0.32), *P* = 0.02), in line with a more frequent detection of the *FCN2* wild-type haplotype GGAACG (−986G > A, −602G > A, −557A > G, −4A > G +6359 C > T and +6424 G > T) in diffuse SSc patients (51% vs. 30%, *P* = 0.03).

MBL levels were significantly elevated in SSc patients with examination findings of pitting, calcinosis and digital ulcers (but not with telangiectasia, pulp atrophy and nailfold capillary dilatation), which was not the case for ficolin-2 levels (Table 4). Of note, higher MBL and ficolin-2 levels were also observed in patients who had received iloprost infusions for Raynaud phenomenon (MBL: median 2.78 (0.84 to 4.31) vs. 0.89 (0.25 to 2.1), *P* = 0.007; ficolin-2: mean 0.71 (0.35) vs. 0.53 (0.34), *P* = 0.05), and MBL high-producing genotypes were also more common in this group (13 (77%) vs. 35 (48%), *P* = 0.06). In contrast, there was no relationship of these lectin pathway proteins with the use of calcium-channel blockers (data not shown).

**Table 4:** **Association of MBL and ficolin-2 with SSc disease manifestations**

**Variables**	**MBL levels (μg/ml), median (IQR)**	***MBL2*** **low-producing genotype, n (%)**	**Ficolin-2 levels (μg/ml), mean (SD)**
*Skin manifestations*			
Calcinosis (yes vs. no)	2.1 (0.7-3.2) vs. 0.8 (0.1-2.1)	2 (7) vs. 16 (27)	0.6 (0.3) vs. 0.6 (0.3)
*P* value	**0.005**	**0.02**	0.9
Pitting (yes vs. no)	1.8 (0.5-3.0) vs. 0.8 (0.1-1.9)	6 (13) vs. 12 (29)	0.6 (0.4) vs. 0.5 (0.3)
*P* value	**0.007**	0.06	0.2
Digital ulcers (yes vs. no)	2.7 (1.0-4.1) vs. 1.0 (0.2-2.3)	1 (8) vs. 17 (22)	0.6 (0.4) vs. 0.6 (0.3)
*P* value	**0.01**	0.2	0.5
*Organ involvement*			
PAH (yes vs. no)	1.7 (0.3-3.8) vs. 1.1 (0.3-2.6)	1 (13) vs. 17 (21)	0.5 (0.2) vs. 0.6 (0.4)
*P* value	0.5	1	0.6
ILD (yes vs. no)	1.7 (0.5-2.9) vs. 0.9 (0.2-2.1)	6 (15) vs. 12 (25)	0.7 (0.3) vs. 0.5 (0.3)
*P* value	0.11	0.3	**0.04**
Renal crisis (yes vs. no)	1.7 (0.2-4.1) vs. 1.1 (0.3-2.6)	1 (25) vs. 17 (20)	1.0 (0.3) vs. 0.6 (0.3)
*P* value	0.6	1	**0.01**
Bowel dysmotility (yes vs. no)	3.1 (0.8-4.2) vs. 1.1 (0.3-2.4)	1 (11) vs. 17 (21)	0.6 (0.3) vs. 0.4 (0.2)
*P* value	0.14	0.7	**0.04**

ILD, interstitial lung disease; IQR, interquartile range; MBL, mannose-binding lectin; PAH, pulmonary arterial hypertension; SD, standard deviation; SSc, systemic sclerosis.

**Figure 2:**
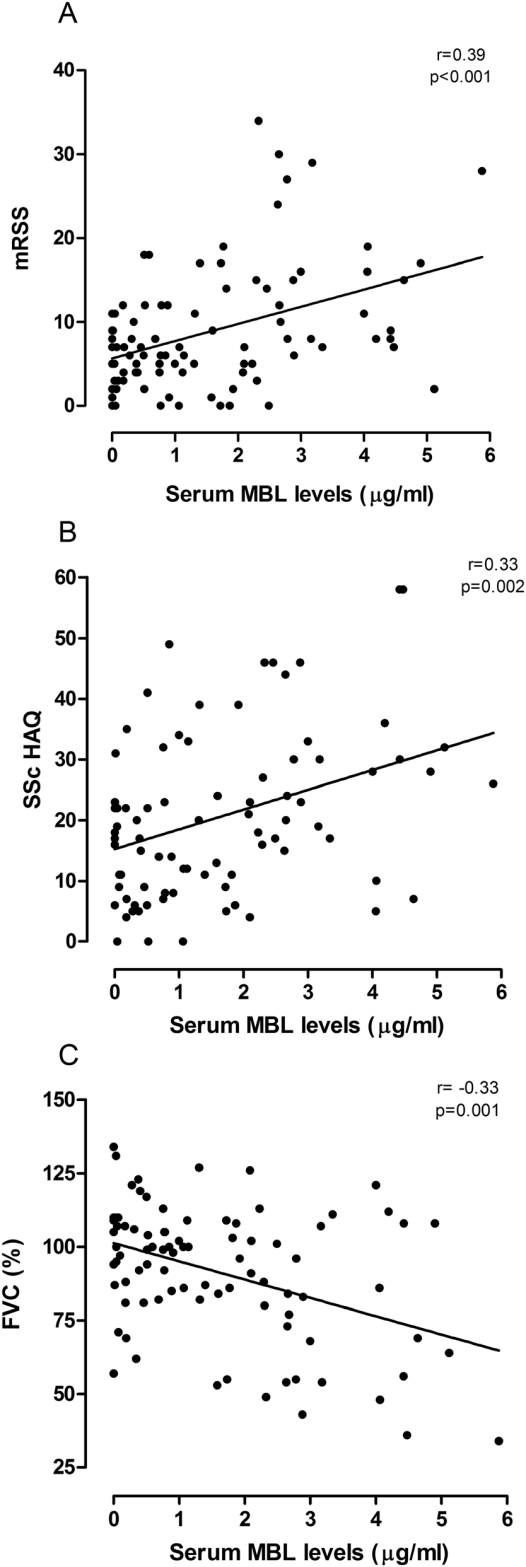
**Correlation of serum MBL levels with activity and extent of disease in SSc cases. (A)** Correlation with extent of skin involvement as assessed by the modified Rodnan skin score (mRSS). **(B)** Correlation with extent of functional disability as assessed by the Scleroderma Health Assessment Questionnaire (SSc HAQ). **(C)** Correlation with extent of pulmonary involvement as assessed by forced vital capacity (FVC, % predicted). MBL, mannose-binding lectin; SSc, systemic sclerosis.

In keeping with a potential association of MBL with certain SSc disease manifestations, MBL levels also correlated with the mRSS and the SSc HAQ score (Figure 2A, B), again supported by genotypic data showing higher mRSS and SSc HAQ scores in cases with wild-type *MBL2* genotypes vs. intermediate-producing vs. low-producing genotypes, respectively (mean mRSS score (SD) 11 (9) vs. 7 (5) vs. 5 (3), *P* <0.01; mean SSc HAQ score 23 (14) vs. 18 (15) vs. 16 (8), *P* = 0.07). After adjustment for sex, age, presence of ILD and PAH, MBL levels remained independently associated with the degree of skin involvement as measured by the mRSS, and with the degree of functional impairment as assessed by the SSc HAQ. The average mRSS and SSc HAQ score increased by 1.7 and 3.6 for every 1 μg/mL increase in MBL serum levels (standard error 0.46 and 0.91, 95% CI 0.73 to 2.56 and 1.63 to 5.24, respectively, *P* <0.001 for both analyses), respectively. In contrast, ficolin-2 levels did not correlate with the mRSS and SSc HAQ. The correlation of mRSS and MBL levels was even stronger when limiting the analysis to diffuse SSc patients (r = 0.48, *P* = 0.02). MBL levels but not ficolin-2 levels correlated with the EUSTAR Systemic Sclerosis Activity Score in diffuse SSc patients (r = 0.49, *P* = 0.02).

There was no association of autoantibodies, levels of inflammatory markers (CRP, ESR) and complement C3/C4 with MBL/ficolin-2 levels or genotpyes (data not shown). In addition, neither MBL nor ficolin-2 levels or genotypes correlated with disease duration in the whole cohort, or when analysis was limited to diffuse SSc patients or those with a disease duration of <5 years vs. ≥5 years (data not shown).

### Association of MBL and ficolin-2 with SSc organ involvement

Higher ficolin-2 levels were associated with bowel dysmotility, ILD and a history of scleroderma renal crisis (but not PAH) with a similar non-significant trend in MBL levels (Table 4). In line, the *FCN2* wild-type haplotype GGAACG was encountered more frequently in patients with ILD (50% vs. 26%, *P* = 0.02).

Regarding ILD, MBL and ficolin-2 levels were inversely correlated with forced vital capacity (FVC) in the whole cohort (Figure 2C), and patients with low-producing *MBL2* genotypes had significantly higher FVC values (mean 103 (20), vs. 89 (22), *P* = 0.02). The correlation of MBL with FVC was even more pronounced when limiting the analysis to diffuse SSc patients (r = −0.55, *P* = 0.008). In addition, higher MBL and ficolin-2 levels were observed in SSc patients with a reduced FVC <70% (median MBL levels 2.88 (1.66 to 4.45) vs. 0.85 (0.18 to 2.0), *P* = 0.001; mean ficolin-2 levels 0.73 (0.39) vs. 0.52 (0.32), *P* = 0.02). Of note, all patients with a FVC <50% had a high-producing *MBL2* genotype. However, MBL and ficolin-2 levels did not correlate with diffusion capacity of the lung for carbon monoxide or six-minute walk distance (data not shown).

Regarding PAH, we observed significantly higher median MBL levels in SSc patients with World Health Organization (WHO) functional class III/IV compared to I/II (2.39 (1.13 to 3.12) vs. 0.81 (0.19 to 2.24), *P* = 0.02) in accordance with a higher frequency of *MBL2* high-producing genotypes (15 (75%) vs. 33 (47%), *P* = 0.03) in the same group. There was no difference in MBL or ficolin-2 polymorphisms/levels with respect to the use of pulmonary vasodilator therapy (data not shown).

Patients currently on immunosuppressive medication had higher median MBL (2.10 (0.80 to 3.13) vs. 0.78 (0.10 to 2.13, *P* = 0.005) and mean ficolin-2 levels (0.67 (0.36) vs. 0.52 (0.32), *P* = 0.05), which was in agreement with *MBL2* genetics (high-producing genotypes: 21(75%) vs. 24 (45), *P* = 0.007).

## Discussion

SSc is a complex and heterogeneous disease characterized by fibrosis, vascular and immune dysfunction. Data from several candidate gene [3] and genome-wide association studies [33-35] (GWAS) suggest that genetic factors may influence not only SSc susceptibility but also the predisposition to develop distinct clinical phenotypes such as limited or diffuse SSc. Given the role of the lectin pathway of complement in general, and MBL in particular in tissue damage related to vasculopathy *in vitro* and *in vivo*, we sought to determine a potential link between the lectin pathway and the predisposition to and severity of SSc in a well-characterized Australian SSc cohort.

While MBL levels and frequency of *MBL* and *FCN2* polymorphisms were similar in controls and the entire SSc cohort, our preliminary data indicate that MBL deficiency (and potentially *FCN2* mutations) might be a protective factor for the development of diffuse SSc. This finding is not necessarily in contradiction to previous GWAS, which have not identified MBL or ficolin-2 as susceptibility genes, as limitations of GWAS include their single-variant-centred approach and lack of phenotypic data. In the case of MBL this might be important, as MBL levels are the result of a complex interplay between several genetic variants in the promoter and exon region [13] and physiologic variables such as thyroid [36] and liver function [37]. With a much weaker genotypic/phenotypic relationship, this might be even more relevant in the case of ficolin-2 [14]. As our sample size was limited, future studies are needed to clarify the association of MBL with diffuse SSc.

MBL deficiency was not associated with anti-Scl-70 autoantibodies commonly detected in diffuse SSc patients, which points to a pathogenesis of MBL-mediated tissue damage that is independent of autoantibody involvement. While not investigated in SSc experimental models, evidence from comprehensive animal and human studies suggests that MBL-mediated damage in I/R injury is caused by binding of MBL to IgM on stressed endothelial and parenchymal cells causing apoptosis [38], activation of the complement and the coagulation cascade (via MASP-1) and recruitment of inflammatory cells [39]. Assuming this analogy, we propose a similar pathogenesis in chronic cutaneous microvascular injury in SSc patients. Previous studies have demonstrated decreased expression of endothelial complement regulatory proteins on the one hand [40] and activation of downstream complement components on the other hand [41] in skin biopsies of SSc patients, even in non-involved skin. However, it remains to be determined if the lectin pathway is primarily responsible for the observed cutaneous complement activation in SSc.

Additionally, we have investigated the association of MBL and ficolin-2 with SSc disease manifestations and severity. In agreement with our *a priori* hypothesis, MBL levels were significantly higher in patients with diffuse SSc vs. limited/sine SSc patients, in patients with vascular disease manifestations, and in patients on immunosuppressive medication or with a history of iloprost infusions for Raynaud phenomenon. The latter may be regarded as a surrogate for more severe disease manifestations as SSc patients with more severe organ disease or digital ulcers are more likely to receive immunosuppressive medication and iloprost infusions, respectively. In addition, MBL levels correlated with extent of skin involvement as assessed by the mRSS and with functional disability/impairment as evaluated by the SSc HAQ. Our results were also supported by genetic data with *MBL2* polymorphisms being rare in patients with diffuse SSc, extensive skin involvement and higher disease activity. The association of MBL levels with skin involvement and disease activity was even more pronounced in diffuse SSc patients, lending support to the hypothesis of SSc being a heterogenous disease with different genetic variants being implicated in specific SSc subtypes [42]. In summary, MBL deficiency seems to be a protective factor for the development of peripheral vascular and skin manifestations in SSc, with less striking results for ficolin-2.

Regarding organ involvement, we have observed significant associations of higher ficolin-2 levels in SSc patients with pulmonary, bowel and renal involvement with a similar non-significant trend in the case of MBL levels. In addition, an inverse correlation of ficolin-2 and MBL levels with FVC was observed, which was again more pronounced in diffuse SSc patients. This is of importance, as FVC is a validated predictor of long-term progression of SSc-related ILD [43]. Recent evidence has identified a central role of stressed or damaged endothelial cells in the development of interstitial lung disease through several different mechanisms, for example activation of the immune system [44]. Interestingly, over a decade ago Collard *et al*. [45] demonstrated binding of MBL to endothelial cells immediately after oxidative stress with subsequent activation of the lectin pathway of complement, which could be prevented by anti-MBL monoclonal antibodies. Independent of downstream complement activation, binding of MBL to stressed endothelial cells might also induce platelet activation and amplification of the coagulation cascade [11,12]. Our results support the hypothesis that endothelial cell dysfunction may induce pulmonary inflammation and fibrosis and that this process might be partially mediated by MBL and ficolin-2.

The fact that MBL and ficolin-2 levels did not correlate with inflammatory markers or complement activity as measured by C3 and C4 levels points to a distinctive response of the lectin pathway in SSc rather than a collective, non-specific proinflammatory immune reaction to endothelial stress. Further studies are needed to identify targets and mechanisms that might be involved in MBL-mediated vasculopathy and fibrosis in affected organs in SSc.

The discovery of MBL and ficolin-2 being associated with specific clinical manifestations in SSc patients might lead to new insights regarding pathogenesis and may facilitate the development of novel therapies that can be targeted to specific subsets of SSc patients. Our results draw attention to MBL and the lectin pathway as a promising target for reducing vascular injury in SSc patients. In fact, a powerful inhibitor of the lectin pathway that binds to and inhibits MBL and MASP-2 already exists. Recombinant human C1 inhibitor (rhC1INH) is a multiple-action-multiple-target inhibitor that not only interferes with the lectin pathway but also with C1 of the classical pathway and the coagulation system [46]. Administration of rhC1INH considerably ameliorated tissue damage in experimental renal and cerebral I/R injury [18,47]. However, data on chronic vascular injury as seen in SSc is lacking.

This is the first study to determine serum levels and polymorphisms of two PRP of the lectin pathway of complement in SSc patients. Previous studies have exclusively examined the activity of the classical or alternative pathway [9,48,49]. In a recent pilot study, Akamata *et al*. determined MBL levels in 63 Japanese SSc patients and 10 healthy controls [50]. *MBL2*/*FCN2* genotypes and ficolin-2 levels were not examined. While confirming our findings of a correlation of MBL levels with extent of skin involvement in diffuse SSc patients, they found no association of MBL levels with pulmonary function tests and clinical manifestations. In addition, MBL levels were similar in diffuse SSc patients and controls. These discrepancies might be related to the sample size of this study (63 SSc cases and 10 controls) and the difference in disease duration and ethnic background [51], as our patients were mainly of Caucasian ethnicity and had on average a longer disease duration.

Despite the precisely characterized prospective cohort, the present study has limitations including the *post hoc* analysis of a limited number of PRRs of the lectin pathway, and the inclusion of patients with mainly late SSc making the cohort prone to survival bias. Indeed, one could argue that higher MBL/ficolin-2 levels are actually protective, if SSc patients with MBL deficiency or low ficolin-2 levels had died early in the disease. However, MBL and ficolin-2 levels did not correlate with disease duration and were similar in SSc patients with disease duration of <5 years vs. ≥5 years in our cohort (data not shown). In addition, of the only two patients that had died since the last annual visit and were included in this analysis, both had high MBL levels (>2.0 μg/mL). On the other hand, the inclusion of patients with long-standing disease offered the advantage to investigate the association of lectin pathway proteins with advanced/late disease manifestations.

We could not determine whether MBL and ficolin-2 have a similar impact early and late in the disease. Ideally, future studies should include additional lectin pathway proteins like ficolin-1/-3 and MASP-1/-2, and evaluate the impact of the lectin pathway on disease manifestations over time. For example, it would be useful to determine if initial MBL and fioclin-2 levels can be used as a predictor for progression of SSC-related ILD as measured by FVC. Although our analysis of the importance of MBL and ficolin-2 in SSc is the largest to date, its significance is limited due to a small sample size. Nevertheless, validity of our results is supported by agreement of phenotypic and genotypic data, in particular with respect to MBL. Pre-analytic issues precluded comparison of ficolin-2 levels in cases and controls [28,29]. It remains speculative, if minor variations in *FCN2* haplotypes and polymorphisms observed in cases and controls translate into a significant difference in ficolin-2 levels, as the ficolin-2 genotypic/phenotypic relationship is much weaker compared to MBL [14]. Lastly, four patients with MCTD were analyzed together with SSc patients as they had been included in the Australian Scleroderma Cohort Study. Subsequent studies investigating the lectin pathway of complement in SSc should ideally only include individuals with diffuse and limited SSc disease excluding MCTD patients.

## Conclusions

In conclusion, our preliminary data suggest a strong association of high MBL and to a lesser extent ficolin-2 levels with certain disease manifestations of SSc. While not being able to demonstrate a causal relationship our findings support the concept of a potential contribution of the lectin pathway to chronic vascular injury and subsequent fibrosis in SSc. If confirmed in future studies, strategies focused on inhibition of MBL and the lectin pathway might provide an additional targeted therapy to supplement the limited therapeutic armamentarium currently available for treatment of patients with SSc.

## Additional file

Additional file 1: Table S1. Taqman genotyping assay details (Life Technologies, Mulgrave, VIC, Australia).

## Abbreviations

CT: computed tomography
CRP: C-reactive protein
ELISA: enzyme-linked immunosorbent assay
ESR: erythrocyte sedimentation rate
FVC: forced vital capacity
GWAS: genome-wide association studies
IgG: immunoglobulin G
I/R: ischemia/reperfusion
ILD: interstitial lung disease
IQR: interquartile range
MASP: mannose-binding lectin-associated serine-protease
MBL: mannose-binding lectin
MCTD: mixed connective tissue disease
mRSS: modified Rodnan skin score
PAH: pulmonary arterial hypertension
PRR: pattern recognition receptor
SD: standard deviation
SSc HAQ: Scleroderma Health Assessment Questionnaire
SSc: systemic sclerosis

## References

[1] Elhai M, Meune C, Avouac J, Kahan A, Allanore Y (2012). Trends in mortality in patients with systemic sclerosis over 40 years: a systematic review and meta-analysis of cohort studies. Rheumatology.

[2] LeRoy EC, Black C, Fleischmajer R, Jablonska S, Krieg T, Medsger TA, Rowell N, Wollheim F (1988). Scleroderma (systemic sclerosis): classification, subsets and pathogenesis. J Rheumatol.

[3] Martin JE, Bossini-Castillo L, Martin J (2012). Unraveling the genetic component of systemic sclerosis. Hum Genet.

[4] Dinkler M (1891). Zur Lehre von der Sklerodermie. Deutsch Arch Klin Med.

[5] Matucci-Cerinic M, Kahaleh B, Wigley FM (2013). Review: evidence that systemic sclerosis is a vascular disease. Arthritis Rheum.

[6] Gabrielli A, Avvedimento EV, Krieg T (2009). Scleroderma. N Engl J Med.

[7] Tyndall AJ, Bannert B, Vonk M, Airo P, Cozzi F, Carreira PE, Bancel DF, Allanore Y, Muller-Ladner U, Distler O, Iannone F, Pellerito R, Pileckyte M, Miniati I, Ananieva L, Gurman AB, Damjanov N, Mueller A, Valentini G, Riemekasten G, Tikly M, Hummers L, Henriques MJ, Caramaschi P, Scheja A, Rozman B, Ton E, Kumánovics G, Coleiro B, Feierl E (2010). Causes and risk factors for death in systemic sclerosis: a study from the EULAR Scleroderma Trials and Research (EUSTAR) database. Ann Rheum Dis.

[8] Benbassat C, Schlesinger M, Luderschmidt C, Valentini G, Tirri G, Shoenfeld Y (1993). The complement system and systemic sclerosis. Immunol Res.

[9] Hudson M, Walker JG, Fritzler M, Taillefer S, Baron M (2007). Hypocomplementemia in systemic sclerosis–clinical and serological correlations. J Rheumatol.

[10] Kjaer TR, Thiel S, Andersen GR (2013). Toward a structure-based comprehension of the lectin pathway of complement. Mol Immunol.

[11] Gulla KC, Gupta K, Krarup A, Gal P, Schwaeble WJ, Sim RB, O’Connor CD, Hajela K (2010). Activation of mannan-binding lectin-associated serine proteases leads to generation of a fibrin clot. Immunology.

[12] La Bonte LR, Pavlov VI, Tan YS, Takahashi K, Takahashi M, Banda NK, Zou C, Fujita T, Stahl GL (2012). Mannose-binding lectin-associated serine protease-1 is a significant contributor to coagulation in a murine model of occlusive thrombosis. J Immunol.

[13] Garred P, Larsen F, Seyfarth J, Fujita R, Madsen HO (2006). Mannose-binding lectin and its genetic variants. Genes Immun.

[14] Kilpatrick DC, St Swierzko A, Matsushita M, Domzalska-Popadiuk I, Borkowska-Klos M, Szczapa J, Cedzynski M (2013). The relationship between FCN2 genotypes and serum ficolin-2 (L-ficolin) protein concentrations from a large cohort of neonates. Hum Immunol.

[15] Davies EJ, Snowden N, Hillarby MC, Carthy D, Grennan DM, Thomson W, Ollier WE (1995). Mannose-binding protein gene polymorphism in systemic lupus erythematosus. Arthritis Rheum.

[16] Schoos MM, Munthe-Fog L, Skjoedt MO, Ripa RS, Lonborg J, Kastrup J, Kelbaek H, Clemmensen P, Garred P (2013). Association between lectin complement pathway initiators, C-reactive protein and left ventricular remodeling in myocardial infarction-a magnetic resonance study. Mol Immunol.

[17] Cervera A, Planas AM, Justicia C, Urra X, Jensenius JC, Torres F, Lozano F, Chamorro A (2010). Genetically-defined deficiency of mannose-binding lectin is associated with protection after experimental stroke in mice and outcome in human stroke. PLoS One.

[18] Gesuete R, Storini C, Fantin A, Stravalaci M, Zanier ER, Orsini F, Vietsch H, Mannesse ML, Ziere B, Gobbi M, De Simoni MG (2009). Recombinant C1 inhibitor in brain ischemic injury. Ann Neurol.

[19] Walsh MC, Bourcier T, Takahashi K, Shi L, Busche MN, Rother RP, Solomon SD, Ezekowitz RA, Stahl GL (2005). Mannose-binding lectin is a regulator of inflammation that accompanies myocardial ischemia and reperfusion injury. J Immunol.

[20] Schwaeble WJ, Lynch NJ, Clark JE, Marber M, Samani NJ, Ali YM, Dudler T, Parent B, Lhotta K, Wallis R, Farrar CA, Sacks S, Lee H, Zhang M, Iwaki D, Takahashi M, Fujita T, Tedford CE, Stover CM (2011). Targeting of mannan-binding lectin-associated serine protease-2 confers protection from myocardial and gastrointestinal ischemia/reperfusion injury. Proc Natl Acad Sci U S A.

[21] Orsini F, Villa P, Parrella S, Zangari R, Zanier ER, Gesuete R, Stravalaci M, Fumagalli S, Ottria R, Reina JJ, Paladini A, Micotti E, Ribeiro-Viana R, Rojo J, Pavlov VI, Stahl GL, Bernardi A, Gobbi M, De Simoni MG (2012). Targeting mannose-binding lectin confers long-lasting protection with a surprisingly wide therapeutic window in cerebral ischemia. Circulation.

[22] Trendelenburg M, Theroux P, Stebbins A, Granger C, Armstrong P, Pfisterer M (2010). Influence of functional deficiency of complement mannose-binding lectin on outcome of patients with acute ST-elevation myocardial infarction undergoing primary percutaneous coronary intervention. Eur Heart J.

[23] Osthoff M, Katan M, Fluri F, Schuetz P, Bingisser R, Kappos L, Steck AJ, Engelter ST, Mueller B, Christ-Crain M, Trendelenburg M (2011). Mannose-binding lectin deficiency is associated with smaller infarction size and favorable outcome in ischemic stroke patients. PLoS One.

[24] Masi AT, Subcommittee for scleroderma criteria of the American Rheumatism Association Diagnostic and Therapeutic Criteria Committee (1980). Preliminary criteria for the classification of systemic sclerosis (scleroderma). Arthritis Rheum.

[25] LeRoy EC, Medsger TA (2001). Criteria for the classification of early systemic sclerosis. J Rheumatol.

[26] Alarcon-Segovia D, Cardiel MH (1989). Comparison between 3 diagnostic criteria for mixed connective tissue disease. Study of 593 patients. J Rheumatol.

[27] Minchinton RM, Dean MM, Clark TR, Heatley S, Mullighan CG (2002). Analysis of the relationship between mannose-binding lectin (MBL) genotype, MBL levels and function in an Australian blood donor population. Scand J Immunol.

[28] Hein E, Bay JT, Munthe-Fog L, Garred P (2013). Ficolin-2 reveals different analytical and biological properties dependent on different sample handling procedures. Mol Immunol.

[29] Brady AM, Spencer BL, Falsey AR, Nahm MH (2014). Blood collection tubes influence serum ficolin-1 and ficolin-2 levels. Clin Vaccine Immunol.

[30] Osthoff M, Au Yong HM, Dean MM, Eisen DP (2013). Significance of mannose-binding lectin deficiency and nucleotide-binding oligomerization domain 2 polymorphisms in staphylococcus aureus bloodstream infections: a case–control study. PLoS One.

[31] Eisen DP, Dean MM, Boermeester MA, Fidler KJ, Gordon AC, Kronborg G, Kun JF, Lau YL, Payeras A, Valdimarsson H, Brett SJ, Ip WK, Mila J, Peters MJ, Saevarsdottir S, van Till JW, Hinds CJ, McBryde ES (2008). Low serum mannose-binding lectin level increases the risk of death due to pneumococcal infection. Clin Infect Dis.

[32] Ouf EA, Ojurongbe O, Akindele AA, Sina-Agbaje OR, Van Tong H, Adeyeba AO, Kremsner PG, Kun JF, Velavan T (2012). Ficolin-2 levels and FCN2 genetic polymorphisms as a susceptibility factor in schistosomiasis. J Infect Dis.

[33] Zhou X, Lee JE, Arnett FC, Xiong M, Park MY, Yoo YK, Shin ES, Reveille JD, Mayes MD, Kim JH, Song R, Choi JY, Park JA, Lee YJ, Lee EY, Song YW, Lee EB (2009). HLA-DPB1 and DPB2 are genetic loci for systemic sclerosis: a genome-wide association study in Koreans with replication in North Americans. Arthritis Rheum.

[34] Radstake TR, Gorlova O, Rueda B, Martin JE, Alizadeh BZ, Palomino-Morales R, Coenen MJ, Vonk MC, Voskuyl AE, Schuerwegh AJ, Broen JC, van Riel PL, van't Slot R, Italiaander A, Ophoff RA, Riemekasten G, Hunzelmann N, Simeon CP, Ortego-Centeno N, González-Gay MA, González-Escribano MF, Airo P, van Laar J, Herrick A, Worthington J, Hesselstrand R, Smith V, de Keyser F, Houssiau F, Spanish Scleroderma Group (2010). Genome-wide association study of systemic sclerosis identifies CD247 as a new susceptibility locus. Nat Genet.

[35] Allanore Y, Saad M, Dieude P, Avouac J, Distler JH, Amouyel P, Matucci-Cerinic M, Riemekasten G, Airo P, Melchers I, Hachulla E, Cusi D, Wichmann HE, Wipff J, Lambert JC, Hunzelmann N, Tiev K, Caramaschi P, Diot E, Kowal-Bielecka O, Valentini G, Mouthon L, Czirják L, Damjanov N, Salvi E, Conti C, Müller M, Müller-Ladner U, Riccieri V, Ruiz B (2011). Genome-wide scan identifies TNIP1, PSORS1C1, and RHOB as novel risk loci for systemic sclerosis. PLoS Genet.

[36] Potlukova E, Jiskra J, Freiberger T, Limanova Z, Zivorova D, Malickova K, Springer D, Grodecka L, Antosova M, Telicka Z, Pesickova SS, Trendelenburg M (2010). The production of mannan-binding lectin is dependent upon thyroid hormones regardless of the genotype: a cohort study of 95 patients with autoimmune thyroid disorders. Clin Immunol.

[37] Altorjay I, Vitalis Z, Tornai I, Palatka K, Kacska S, Farkas G, Udvardy M, Harsfalvi J, Dinya T, Orosz P, Lombay B, Par G, Par A, Csak T, Osztovits J, Szalay F, Csepregi A, Lakatos PL, Papp M (2010). Mannose-binding lectin deficiency confers risk for bacterial infections in a large Hungarian cohort of patients with liver cirrhosis. J Hepatol.

[38] van der Pol P, Schlagwein N, van Gijlswijk DJ, Berger SP, Roos A, Bajema IM, de Boer HC, de Fijter JW, Stahl GL, Daha MR, van Kooten C (2012). Mannan-binding lectin mediates renal ischemia/reperfusion injury independent of complement activation. Am J Transplant.

[39] Osthoff M, Trendelenburg M (2013). Impact of mannose-binding lectin deficiency on radiocontrast-induced renal dysfunction. Biomed Res Int.

[40] Venneker GT, van den Hoogen FH, Boerbooms AM, Bos JD, Asghar SS (1994). Aberrant expression of membrane cofactor protein and decay-accelerating factor in the endothelium of patients with systemic sclerosis. A possible mechanism of vascular damage. Lab Invest.

[41] Sprott H, Muller-Ladner U, Distler O, Gay RE, Barnum SR, Landthaler M, Scholmerich J, Lang B, Gay S (2000). Detection of activated complement complex C5b-9 and complement receptor C5a in skin biopsies of patients with systemic sclerosis (scleroderma). J Rheumatol.

[42] Gorlova O, Martin JE, Rueda B, Koeleman BP, Ying J, Teruel M, Diaz-Gallo LM, Broen JC, Vonk MC, Simeon CP, Alizadeh BZ, Coenen MJ, Voskuyl AE, Schuerwegh AJ, van Riel PL, Vanthuyne M, van't Slot R, Italiaander A, Ophoff RA, Hunzelmann N, Fonollosa V, Ortego-Centeno N, González-Gay MA, García-Hernández FJ, González-Escribano MF, Airo P, van Laar J, Worthington J, Hesselstrand R, Smith V (2011). Identification of novel genetic markers associated with clinical phenotypes of systemic sclerosis through a genome-wide association strategy. PLoS Genet.

[43] Furst D, Khanna D, Matucci-Cerinic M, Clements P, Steen V, Pope J, Merkel P, Foeldvari I, Seibold J, Pittrow D, Polisson R, Strand V (2007). Systemic sclerosis - continuing progress in developing clinical measures of response. J Rheumatol.

[44] Leach HG, Chrobak I, Han R, Trojanowska M (2013). Endothelial cells recruit macrophages and contribute to a fibrotic milieu in bleomycin lung injury. Am J Respir Cell Mol Biol.

[45] Collard CD, Vakeva A, Morrissey MA, Agah A, Rollins SA, Reenstra WR, Buras JA, Meri S, Stahl GL (2000). Complement activation after oxidative stress: role of the lectin complement pathway. Am J Pathol.

[46] Davis AE, Cai S, Liu D (2007). C1 inhibitor: biologic activities that are independent of protease inhibition. Immunobiology.

[47] Castellano G, Melchiorre R, Loverre A, Ditonno P, Montinaro V, Rossini M, Divella C, Battaglia M, Lucarelli G, Annunziata G, Palazzo S, Selvaggi FP, Staffieri F, Crovace A, Daha MR, Mannesse M, van Wetering S, Paolo Schena F, Grandaliano G (2010). Therapeutic targeting of classical and lectin pathways of complement protects from ischemia-reperfusion-induced renal damage. Am J Pathol.

[48] Senaldi G, Lupoli S, Vergani D, Black CM (1989). Activation of the complement system in systemic sclerosis. Relationship to clinical severity. Arthritis Rheum.

[49] Foocharoen C, Distler O, Becker M, Muller-Ladner U, von Muhlen C, Leuchten N, Walker UA (2012). Clinical correlations of hypocomplementaemia in systemic sclerosis: an analysis of the EULAR Scleroderma Trial and Research group (EUSTAR) database. Scand J Rheumatol.

[50] Akamata K, Asano Y, Aozasa N, Noda S, Taniguchi T, Takahashi T, Ichimura Y, Toyama T, Sumida H, Kuwano Y, Yanaba K, Tada Y, Sugaya M, Kadono T, Sato S (2014). Serum levels of mannose-binding lectin in systemic sclerosis: a possible contribution to the initiation of skin sclerosis in the diffuse cutaneous subtype. Eur J Dermatol.

[51] Reveille JD (2003). Ethnicity and race and systemic sclerosis: how it affects susceptibility, severity, antibody genetics, and clinical manifestations. Curr Rheumatol Rep.

