# Supplementary material for: Potential role of the lectin pathway of complement in the pathogenesis and disease manifestations of systemic sclerosis: a case-control and cohort study
Source: Arthritis Res Ther. 2014 Nov 18;16(6):480. doi: 10.1186/s13075-014-0480-6 (PMC4264552; doi:10.1186/s13075-014-0480-6)
Supplement: Additional file 1: Table S1. — Taqman genotyping assay details (Life Technologies, Mulgrave, VIC, Australia). [file 13075_2014_480_MOESM1_ESM.pdf]

Table S1: Taqman genotyping assay details (Life Technologies, Australia)

		SNP			
**Nucleotid Change**	**Localization**	**Database ID**	**Assay Reference**	**Primers**	**Fluorescent Probes**
MBL2 -X/Y	10q11.2-q21	rs7096206	C__27858274_10		
MBL2 -B (codon 54)	10q11.2-q21	rs1800450	C___2336609_20		
MBL2 -C (codon 57)	10q11.2-q21	rs1800451	C___2336608_20		
MBL2 -D (codon 52)	10q11.2-q21	rs5030737	C___2336610_10		
FCN2 -986 (G>A) [1]	9q34	rs3124952		5‘-GGACCTCGGCATCCC-3‘	VIC-ACCTGCCGCCATC
				5‘-CCACCACCGCACCCT-3‘	FAM-CACCTGCTGCCATC
FCN2 -602 (G>A)	9q34	rs3124953	C__27461651_20		
FCN2 -557 (A>G)	9q34	rs3811140	C_____65537_10		
FCN2 -4 (A>G)	9q34	rs17514136	C__25765134_10		
FCN2 +6359 (C>T)	9q34	rs17549193	C__61859150_10		
FCN2 +6424 (G>T)	9q34	rs7851696	C__29220549_20		

Abbreviations: MBL, mannose-binding lectin; FCN2, ficolin-2

Reference:

1. Munthe-Fog L, Hummelshoj T, Hansen BE, Koch C, Madsen HO, Skjodt K, Garred P: **The impact of FCN2 polymorphisms and haplotypes on the Ficolin-2 serum levels**. *Scand J Immunol* 2007, **65**(4):383-392.
